# Richard Bayly Winder, M. D., D. D. S.

**Published:** 1894-09

**Authors:** 


### Obituary.

### RICHARD BAYLY WINDER, M.D., D.D.S.

Died at Baltimore, Md., July 18, 1894, Professor R. B. Winder, Dean of the Baltimore College of Dental Surgery.

He was born July 17, 1828, at Accomac Court-House, then called Drummondtown, on the Eastern Shore of Virginia. He was the eldest born of Nathaniel James Winder and wife, Sarah Upshur Bayly. The family were in direct descent from Sir George Yardley, one of the early governors of Virginia.

Dr. Winder was educated at Princeton and the Virginia University. He married, at an early age, into the Curtis family and settled in Accomac County on one of the old homesteads, called "The Folly," where he resided until 1861. When the war broke out between the North and the South, he was among the first to tender his service and that of his company to the Confederate States. At the close of the war, like the majority of those connected with the "Lost Cause," he returned to his home without sufficient means to live as he had been accustomed to, in luxurious ease and elegance. With characteristic energy he set to work to build for the future, instead of vainly repining at his serious losses and much suffering. It was said of him by the late Governor Henry A. Wise, who had known him from boyhood, that "Dick Winder had lost and suffered more than any man he knew, and with less complaint."

Dr. Winder was married in April, 1869, to Miss Kate Dorsey, of Frederick County, Md. The same year, he graduated in dentistry at the Baltimore College of Dental Surgery. His interest in dental education was a prominent feature of his active life. He organized, in company with others, the Maryland Dental College. This was merged into the Baltimore College of Dental Surgery, of which he eventually became the Dean. His interest thereafter was largely bound up in college work. Professor Winder's ability as an educator was recognized throughout the dental profession, and largely through his influence the Baltimore College prospered as never before. His work was thorough, and his kindness to the young men under his charge was marked in character as it was decided in its influence for good. He was the originator of the idea of a national organization of dental colleges of the United States, and this bore fruit in the formation of the National Association of Dental Faculties, the most powerful organization probably ever established. Through its influence the entire course of dental education has been changed and the standard advanced beyond the anticipations of the most sanguine.

The intimate relations of the writer with Dr. Winder long since led to the conclusion that his genial character, scholarly ability, and progressive tendencies were not as well understood as they should have been. He was earnest in his convictions and faithful to his friends, and in his death the profession has to mourn one who faithfully worked for its best interests to his last hour.

The funeral took place from his residence, 716 Park Avenue, Baltimore, July 21, 1894. J. T.

